# The mammalian proton-coupled peptide cotransporter PepT1: sitting on the transporter–channel fence?

**DOI:** 10.1098/rstb.2008.0139

**Published:** 2008-10-28

**Authors:** David Meredith

**Affiliations:** School of Life Sciences, Oxford Brookes UniversityGipsy Lane, Headington, Oxford OX3 0BP, UK

**Keywords:** epithelia, proton-coupled peptide transport, nutrient uptake, drug absorption, site-directed mutagenesis

## Abstract

The proton-coupled di- and tripeptide transporter PepT1 (SLC15a1) is the major route by which dietary nitrogen is taken up from the small intestine, as well as being the route of entry for important therapeutic (pro)drugs such as the β-lactam antibiotics, angiotensin-converting enzyme inhibitors and antiviral and anti-cancer agents. PepT1 is a member of the major facilitator superfamily of 12 transmembrane domain transporter proteins. Expression studies in *Xenopus laevis* on rabbit PepT1 that had undergone site-directed mutagenesis of a conserved arginine residue (arginine_282_ in transmembrane domain 7) to a glutamate revealed that this residue played a role in the coupling of proton and peptide transport and prevented the movement of non-coupled ions during the transporter cycle. Mutations of arginine_282_ to other non-positive residues did not uncouple proton–peptide cotransport, but did allow additional ion movements when substrate was added. By contrast, mutations to positive residues appeared to function the same as wild-type. These findings are discussed in relation to the functional role that arginine_282_ may play in the way PepT1 operates, together with structural information from the homology model of PepT1 based on the *Escherichia coli* lactose permease crystal structure.

## 1. Introduction

The uptake of di- and tripeptides by the proton-coupled transporters of the SLC15 family is widely accepted to be the major route of dietary nitrogen absorption from the small intestine (via SLC15a1/PepT1) and nitrogen reabsorption from the glomerular filtrate in the renal proximal tubule (via PepT1 and SLC15a2/PepT2) (see Meredith & Boyd 2000 and Daniel & Kottra 2004 for reviews). Moreover, PepT1 holds considerable interest for pharmacologists in being a major route of entry for orally bioavailable compounds, such as the β-lactam antibiotics, angiotensin-converting enzyme inhibitors, antiviral and anti-cancer agents (reviewed in Terada & Inui 2004). Despite not being di- or tripeptides, these therapeutic compounds are peptidomimetics and are transported by virtue of their having a similar three-dimensional shape to endogenous substrates. The ability of PepT1 to carry so many substrates (most, if not all, naturally occurring di- and tripeptides, Vig *et al*. 2006) has stimulated much interest in the binding site of this transporter, which has been modelled by several groups (Bailey *et al*. 2000, 2006; Biegel *et al*. 2005). PepT2 is less well characterized, but is thought to be of higher affinity and to have a narrower substrate range than PepT1, although still accepting a large number of substrates compared with most transporters (Biegel *et al*. 2006).

Most studies on PepT1 have been performed on either the rabbit isoform (Fei *et al*. 1994) or the human isoform (Liang *et al*. 1995). Both are predicted to have 12 transmembrane spanning domains (TMDs), which has largely been confirmed experimentally by epitope mapping (Covitz *et al*. 1998; although see Meredith & Price 2006 for discussion about TMD1). The rabbit PepT1 (707 amino acids) and human PepT1 (708 amino acids) share an overall identity of 81 per cent at the amino acid level, with the major areas of difference being in the large extracellular loop between TMDs 9 and 10, and the intracellular C-terminus. In the absence of a crystal structure, computer (Bolger *et al*. 1998) and homology modelling (Meredith & Price 2006) have been attempted, but only time and experimental testing will tell whether these models are accurate enough to be useful. In the meantime, site-directed mutagenesis has been a useful technique in identifying functionally important residues in PepT1, and one such residue is arginine_282_.

## 2. The intriguing arginine_282_

Arginine_282_ (R282) is located approximately halfway down TMD7, and is either an arginine or a lysine in all of the mammalian PepT1 sequences to date. This high level of conservation of a positively charged residue in a TMD suggested a functional role and this residue has been mutated by site-directed mutagenesis to explore such a role. The first study by Bolger *et al*. (1998) reported that mutation of R282 to an alanine in human PepT1 (R282A-hPepT1) had a modest effect on PepT1 activity when expressed in HEK293 cells, namely no change in affinity and a small decrease of approximately 15 per cent in *V*_max_ when compared with the wild-type hPepT1. TMD7 cysteine-scanning experiments by the same group showed that R282C- and R282E-hPepT1 expressed in HEK293 cells also had reduced transport activity of approximately 80 and 43% of wild-type activity, respectively (Kulkarni *et al*. 2003). At the same time, the effect of an R282E mutant in rabbit PepT1 (R282E-rbPepT1) on the kinetics of peptide transport was being investigated (Meredith 2004). In agreement with the previous work on hPepT1, the R282E-rbPepT1 mutant had an apparently lower *V*_max_ when expressed in *Xenopus laevis* oocytes. However, investigation of kinetic parameters other than *V*_max_ revealed that R282E-rbPepT1 was more subtly affected than would at first appear (Meredith 2004), a finding that was further confirmed in a later, more extensive study (Pieri *et al*. 2008).

In the original study (Meredith 2004), measurement of dipeptide uptake by R282E-rbPepT1 revealed that dipeptide uptake was no longer stimulated when the extracellular pH (pH_out_) of the medium was dropped to 5.5 from pH_out_ 7.4, compared with wild-type where the uptake rate was approximately doubled. There was no change in R282E-rbPepT1 substrate affinity, and there was a faster efflux rate in *trans*-stimulation experiments. These findings were consistent with the simplest explanation for the data, i.e. R282E-rbPepT1 was mediating facilitated diffusion of peptides, rather than the obligatory peptide–proton cotransport of the wild-type rbPepT1. Further support for this hypothesis came from the observation that in contrast to wild-type PepT1, R282E-rbPepT1 could not accumulate the neutral substrate above the intracellular concentration that would be predicted at equilibrium. In experiments that were designed to confirm that R282E-rbPepT1 was indeed behaving as a facilitated diffusion peptide transporter, the oocyte membrane potential was measured in the absence and presence of a substrate. Rather unexpectedly R282E-rbPepT1 gave not only a depolarization in the presence of substrate, but also the depolarization was larger than that of the wild-type transporter. These observations were clearly not consistent with the mutant R282E-rbPepT1 simply transporting neutral dipeptide uncoupled to the movement of ions. Two-electrode voltage clamp of oocytes expressing R282E-rbPepT1 suggested that the current induced in the presence of substrate was due to the movement of cations, and it was proposed that there was a peptide-gated cation pathway (‘channel’) in R282E-rbPepT1 that was not present in the obligatory 1 : 1 proton : neutral dipeptide rbPepT1.

In the subsequent study (Pieri *et al*. 2008), the loss of the proton coupling of R282 mutants was shown to be true, namely for R282D-, R282A- and R282Q-rbPepT1. However, if the amino acid residue at position 282 was kept positive (R282K), or had the potential to be positive (R282H), then proton-coupling appeared normal. When substrate accumulation was studied, not only did R282K and R282H-rbPepT1 show concentrative dipeptide uptake, as expected, but so also did the non-proton stimulated mutants, R282D-, R282A- and R282Q-rbPepT1. This surprising result implied that peptide transport by R282D-, R282A- and R282Q-rbPepT1 was still coupled to the movement of protons, but that the rate-limiting step of transport was not influenced by the imposition of an inwardly directed proton gradient, unlike the wild-type transporter. This was confirmed by the finding that the *trans*-stimulation efflux rates of all mutants except for R282E-rbPepT1 was not different from wild-type rbPepT1. If R282E-rbPepT1 did have a peptide-gated cation conductance in addition to a facilitated diffusion pathway for peptides, one question was whether the movement of ions was conditional on transport. This was addressed using a known non-translocated substrate for PepT1, 4-aminobenzoic acid (4-AMBA, Meredith *et al*. 1998), which despite binding to PepT1 was not able to induce any membrane depolarization in R282E-rbPepT1 expressing oocytes. The hypothesis that the reason R282E-rbPepT1 was not able to cause concentrative uptake of substrate was because the flow of current was collapsing the membrane potential (the major driving force for PepT1-mediated transport, Temple *et al*. 1995) was tested by measuring the current induced in other mutants using two electrode voltage clamp. Despite being able to concentrate substrate intracellularly, R282D- and R282A-rbPepT1 also showed an increased stoichiometry from the normal one proton to one neutral dipeptide (Temple *et al*. 1995), and in fact the magnitude of the increase was slightly larger than for R282E-rbPepT1. The currents that had been measured by two electrode voltage clamp for R282E-rbPepT1 in the original study were small (in the sub-microamp range), and the stoichiometry measured later was of the order of four protons to each peptide at a pH_out_ of 5.5. That protons could be carrying the current was supported by a fall in stoichiometry to approximately two protons per peptide when pH_out_ was increased to 7.4, again demonstrating that the movement of charge required, but was not coupled to, the movement of substrate.

## 3. What role does R282 play in PepT1 function?

As can be seen from the data reviewed above, R282 is playing a complicated role in PepT1 function, and the effect of mutation on transporter function is dependent on the substituted residue. There is good evidence that R282 interacts with a negatively charged aspartate at position 341, which is in TMD8 and predicted to be at approximately the same position in the membrane. In a doubly mutated transporter where the charges have been swapped, i.e. R282E-D341R- and R282D-D341R rbPepT1, normal transport characteristics are seen (Pieri *et al*. 2004, 2008) and this was also true for analogous mutations in the human PepT1 (Kulkarni *et al*. 2007). Strangely, the single D341R-rbPepT1 also behaved as wild-type (Pieri *et al*. 2004), whereas the D341R-hPepT1 had significantly reduced transport (Kulkarni *et al*. 2007), and the reason for this discrepancy between species is not clear.

**Figure 1:**
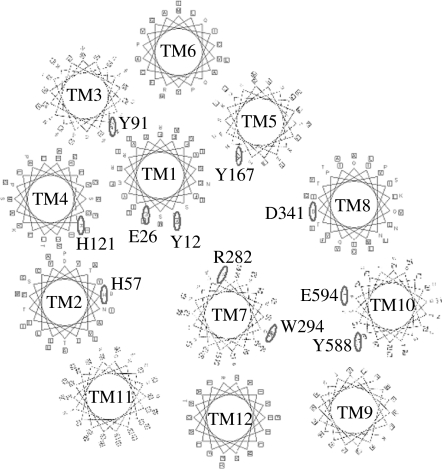
Helical wheel plan of the TMDs in PepT1 showing the putative relative proximity of H57, R282 and D341 in the TMDs 2, 7 and 8, respectively, in the homology model of rabbit PepT1 (modified from Meredith & Price 2006).

Aside from this potential interaction, what else can be deduced about the role of R282? As the only mutant tested that is no longer able to accumulate substrate above the extracellular concentration, it seems logical to assume that peptide movement by R282E-rbPepT1 is no longer coupled to the electrochemical proton gradient. However, the same cannot be true for R282D-rbPepT1, which despite also having a negatively charged amino acid residue at position 282 is able to perform concentrative uptake and so must still be linked to the proton electrochemical gradient despite the uptake rate not being proton stimulated. One explanation for the lack of proton stimulation for the mutant transporters could be that R282E is changing the protein environment of the crucial histidine in TMD2 (H57), long implicated as the residue in PepT1 protonated as the first step in the transport cycle so that it is always protonated (Meredith & Boyd 1995; Temple *et al*. 1996). Support from this comes from the finding that if the rate of transport is normalized to the amount of protein expressed in the oocyte membrane using luminometry (Panitsas *et al*. 2006), then the rate of transport by R282E-rbPepT1 is in fact the same as the wild-type at pH_out_ 5.5, but unlike the wild-type is not slower at pH_out_ 7.4. At pH_out_ 7.4, it has been proposed that the rate limiting step of the transporter kinetic cycle is the protonation of the carrier (Temple *et al*. 1996), and so if in R282E-rbPepT1 H57 was always protonated, the rate-limiting step would be the return of the empty carrier at pH_out_ 7.4 as it is at pH_out_ 5.5 (Temple *et al*. 1996). Thus, there would be no change in transport rate between pH_out_ 5.5 and 7.4, as observed. In the *ab initio* computer modelling of PepT1, TMDs 2 and 7 were not close (Bolger *et al*. 1998); however, the more recent homology modelling suggests that they would be adjacent (Meredith & Price 2006; figure 1). However, the homology model is predicated on the assumption that all members of the major facilitator superfamily (MFS), a large family of over 1000 proteins identified by signature motifs as transporters (Saier *et al*. 2006), will have the same overall three-dimensional structure, as suggested by Abramson *et al*. (2004). Continued advances in membrane protein crystallography will be necessary to prove whether this assumption is valid.

The second difference between R282E-rbPepT1 and the other mutants is that the former is the only mutant tested that cannot concentrate peptide substrate intracellularly above its electrochemical gradient. This suggests that R282E-rbPepT1 is the only mutant for which peptide transport is not coupled to the obligatory cotransport of a proton. This is supported by the stoichiometry data indicating that R282D- and R282A-rbPepT1 had a stoichiometry one higher then R282E-rbPepT1 at both pH_out_ 5.5 and 7.4 (Pieri *et al*. 2008). One possible explanation for this would be that the proton that is widely believed to bind to H57 is the one that is translocated with the peptide substrate: it is not able to dissociate during the transport cycle of R282E-rbPepT1 due to a change induced in the local protein environment that affects the p*K*_a_ of H57 and makes it permanently protonated. The model proposed by Pieri *et al*. suggests that the proton from H57 is transferred to the C-terminal carboxyl group of the zwitterionic peptide substrate, which is then translocated across the membrane. On release this proton dissociates to give a zwitterionic peptide and a free proton in the cytoplasm.

One final difference between wild-type PepT1 and PepT1 mutants lacking a positive charge at position 282 is the movement of additional charge during the transport cycle, with about three extra charges moved in the putatively proton-coupled mutants, and four moved in the non-proton coupled R282E-rbPepT1. While this ion movement is modest compared with the flux that might be expected though a channel, it is nevertheless a significant ‘slippage’ compared with the obligatory one to one coupling of a proton to a neutral dipeptide. One explanation for the extra ion movement could be that the presence of the positively charged R282 residue in the wild-type protein sets up an electrostatic repulsion for protons and other cations that enter the binding site for peptides in the transporter. That there would be space for ions to enter with a substrate seems probable given the large size differences in PepT1 substrates. Thus, during the translocation step when the protein presumably will undergo a substantial conformational change, the positive charge repulsion would prevent ions from passing through the protein unregulated. The lower stoichiometry seen when the proton electrochemical gradient is lower (pH_out_ 7.4 versus 5.5) does suggest that the ion movement is simply electrodiffusive in nature.

## 4. Extrapolation to other transporters

As mentioned above, it has been hypothesized that all members of the MFS will have a similar three-dimensional architecture (Abramson *et al*. 2004). Therefore, it is interesting to look at the mutational analysis of residues of other transporters with regard to the induction of ion conductances not seen in the wild-type. There are examples in the literature of mutations in transporters that induce ion conductances for previously excluded ions, for example, cation channel behaviour in mutants of the chloride–bicarbonate anion exchanger AE1 (Bruce *et al*. 2005). Other mutations may lead to the formation of a channel for (one of) the previously transported ion(s), e.g. in trout AE1 (Martial *et al*. 2006) or the neuronal glutamate transporter EAAC1 (Borre & Kanner 2004). There are also examples of mutations in lactose permease where the lactose transport is uncoupled from proton movement (Kaback *et al*. 2001). However, for both the EAAC1 and the lactose permease mutations the substrate binding affinity is affected, and that is not the case for the PepT1 mutants, suggesting that R282 does not play a role in the peptide substrate binding site.

## 5. Conclusions

R282 plays an intriguing role in PepT1 function. Mutation to anything other than a positively charged residue abolishes the stimulation of transport by an inwardly directed proton electrochemical gradient, yet all mutant PepT1 tested, except R282E-PepT1, can accumulate substrate. The loss of the charged residue at position 282 also seems to be linked to the increased stoichiometry of proton to peptide transport, although at an extra three charges per peptide at pH_out_ 5.5 it is a relatively modest effect. This suggests that rather than a true channel being formed, there is a small slippage of ions during the conformational change in the protein that must occur during the translocation of peptide from one side of the membrane to the other. Further understanding of the structure–function relationship of the PepT1 transporter, including of the substrate binding site, will be invaluable in the future development of orally bioavailable therapeutic compounds.
